# Study of Neutron Radiation Effects in Optical MOS Solid-State Relays

**DOI:** 10.3390/s26165259

**Published:** 2026-08-19

**Authors:** Zijie He, Hao Yu, Chengxiang Han, Zhigang Peng, Baolong Ma, Pei Li, Chaohui He

**Affiliations:** School of Nuclear Science and Technology, Xi’an Jiaotong University, Xi’an 710049, Chinabaolongma@xjtu.edu.cn (B.M.); hechaohui@xjtu.edu.cn (C.H.)

**Keywords:** optical MOS solid-state relay, displacement damage, TCAD simulation, off-state leakage current

## Abstract

Optical MOS solid-state relays provide electrical isolation and favorable switching performance, making them suitable as isolated switching interfaces for optoelectronic sensors used in radiation monitoring systems, high-energy physics accelerator monitoring systems, and aerospace electronic equipment. However, to the best of our knowledge, the reliability of this device under atmospheric-like neutron irradiation has not been systematically investigated. In this study, the neutron-induced degradation characteristics and potential physical mechanisms of a commercial optical MOS solid-state relay were systematically investigated through electrical characterization, Sentaurus TCAD mixed-mode simulation, and post-irradiation annealing experiments. Particular attention was paid to the possible internal coupling relationship between degradation of the internal photoelectric conversion unit and the increase in the off-state leakage current of the output-side power MOSFET.

## 1. Introduction

Solid-state relays are critical components in power systems. With advances in semiconductor technology, optical MOS solid-state relays, which integrate optical isolation with MOSFET technology, have become considerably smaller than traditional solid-state relays. This reduction in size facilitates their integration into complex circuits. Because of their excellent switching characteristics, optical MOS solid-state relays are widely used in power systems and various electronic devices and equipment [1,2,3,4,5,6].

In sensor systems, optical MOS solid-state relays primarily perform interface functions such as electrical isolation, signal switching, and channel selection. Previous studies have used them as isolation switches for microfluidic electrode switching in the control interfaces of biosensing and optoelectronic sensing systems [7]; other studies have employed them as signal conditioning and impedance switching devices in capacitive or electrostatic sensing circuits [8]. Furthermore, in the fields of high-energy physics and nuclear radiation detection, optical MOS solid-state relays can also be used in the bias, readout, or auxiliary control circuits of photomultiplier tubes to achieve isolation and switching control of detection channels [9]. Optical MOS solid-state relays are not only power switching devices but also serve as critical opto-isolated interface devices connecting sensing units, signal conditioning circuits, and control systems.

However, studies on the reliability of optical MOS solid-state relays under complex radiation environments remain relatively limited, and existing reports have mainly focused on gamma-ray total ionizing dose effects and proton irradiation effects. Li Zu’an et al. evaluated a space-grade optical MOS solid-state relay through gamma-ray irradiation experiments and demonstrated its radiation tolerance at a total dose of up to 30 krad [10]. Johnston et al. conducted proton and gamma-ray total dose irradiation experiments on power MOSFET-output optocouplers and systematically investigated proton-induced displacement damage in the light-emitting diode and gamma-ray-induced ionizing damage in the MOSFET [11]. Neitzert H. C. et al. directly compared the proton radiation tolerance of two optoelectronic devices with different structures, namely an optocoupler and an optical MOS solid-state relay [12]. Proton irradiation generally induces both ionizing damage and displacement damage and may also result in synergistic interactions between the two damage mechanisms [13,14]. Consequently, existing proton irradiation results cannot independently reflect the displacement damage induced by neutron irradiation. The degradation characteristics and internal coupling mechanisms of optical MOS solid-state relays in neutron environments therefore require further investigation.

In contrast, relatively few studies have investigated neutron-induced displacement damage in optically isolated interface devices. Mc Marr et al. conducted 14 MeV neutron and ^60^Co gamma-ray irradiation tests on a power MOSFET-output optocoupler, providing an important reference for comparing the effects of neutron-induced displacement damage and gamma-ray-induced ionizing damage on the different functional units of this type of device [15].

Existing studies have primarily focused on the radiation response of individual functional units or have evaluated overall device degradation solely through externally accessible terminal parameters. However, whether changes in the performance of the internal photoelectric conversion unit of an optical MOS solid-state relay can couple to the MOSFET gate node through the internal circuitry and subsequently affect the off-state electrical characteristics of the output stage has not yet been systematically investigated. Therefore, this study focuses on a commercial optical MOS solid-state relay and combines electrical characterization, Sentaurus TCAD mixed-mode simulation, and post-irradiation annealing experiments to systematically analyze the degradation characteristics of its different functional units under neutron irradiation. Particular attention is given to the possible coupling relationship between the degradation of the internal photoelectric conversion unit and the increase in the off-state leakage current of the output-side power MOSFET.

## 2. Materials and Methods

### 2.1. Experimental Devices

A commercial optical MOS solid-state relay was selected as the test device in this study. The device is housed in a standard DIP8 package, with external metal pins providing electrical connections to its internal functional electrodes. According to the device datasheet, this device is designed for control applications in aerospace, spaceflight, and other electronic equipment systems.

**Figure 1:**
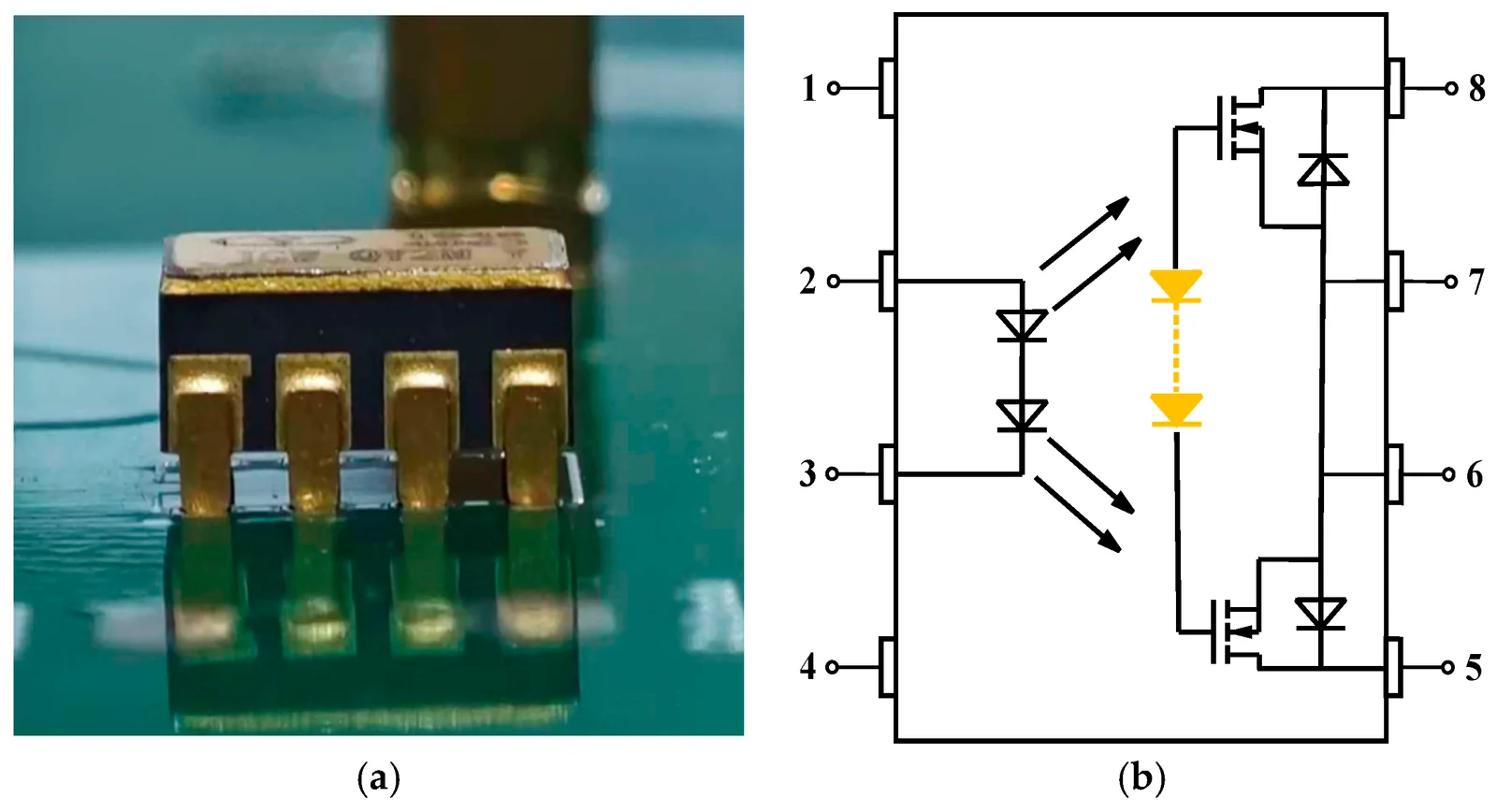
Optical MOS solid-state relay: (**a**) physical diagram of the device; (**b**) internal circuit structure diagram.

Figure 1a,b show a picture and the internal circuit diagram of the investigated optical MOS solid-state relay, respectively. The device mainly consists of an input-side GaAs-based light-emitting diode (GaAs-LED), a Si-based photoelectric conversion unit, and an output-side Si-based power MOSFET. The externally accessible functional electrodes mainly include the anode and cathode of the GaAs-LED, as well as the source and drain of the output MOSFET. According to Table 1, under the DC output configuration, the rated output voltage and rated output current are 60 V and 5 A, respectively. This design is related to the operating principle of this device model, which is triggered by the input-side light-emitting diode. When current flows through the input-stage GaAs-LED, the internal optical path of the device is activated. The photodiode receives the optical signal from the GaAs-LED and generates a voltage high enough to drive the MOSFET’s gate, thereby switching the device from the off state to the on state.

A total of three commercial optical MOS solid-state relays of the same model were selected as test samples in this study. Before irradiation, the initial electrical characteristics of each device were measured individually. The three devices were then irradiated separately at neutron fluences of 7.0 × 10^10^ n/cm^2^, 1.0 × 10^11^ n/cm^2^, and 3.9 × 10^12^ n/cm^2^, with one device assigned to each fluence level. A paired comparison approach was adopted for data analysis, in which the post-irradiation electrical characteristics of each device were compared with those measured for the same device before irradiation. Using an independent device for each neutron fluence level avoided the cumulative influence of preceding irradiation steps on the results obtained at subsequent fluence levels, which could occur if the same device were subjected to stepwise cumulative irradiation.

### 2.2. Experimental Details

To investigate the effects of neutron irradiation on the electrical performance of optical MOS solid-state relays, irradiation experiments were conducted at the Atmospheric Neutron Irradiation Spectrometer (ANIS) of the China Spallation Neutron Source (CSNS) in Dongguan, China. ANIS is a 40 m long ground-based facility that entered operation in 2022 and provides a broad neutron spectrum closely resembling that of atmospheric neutrons [16,17]. The beamline is equipped with a neutron shutter, a primary neutron flight tube, a neutron expander, a neutron flux controller, two collimators, a clearing magnet, a secondary neutron flight tube, neutron filters, and beamline shielding. Most of these components operate under vacuum to minimize air activation induced by high-energy particles [18].

**Figure 2:**
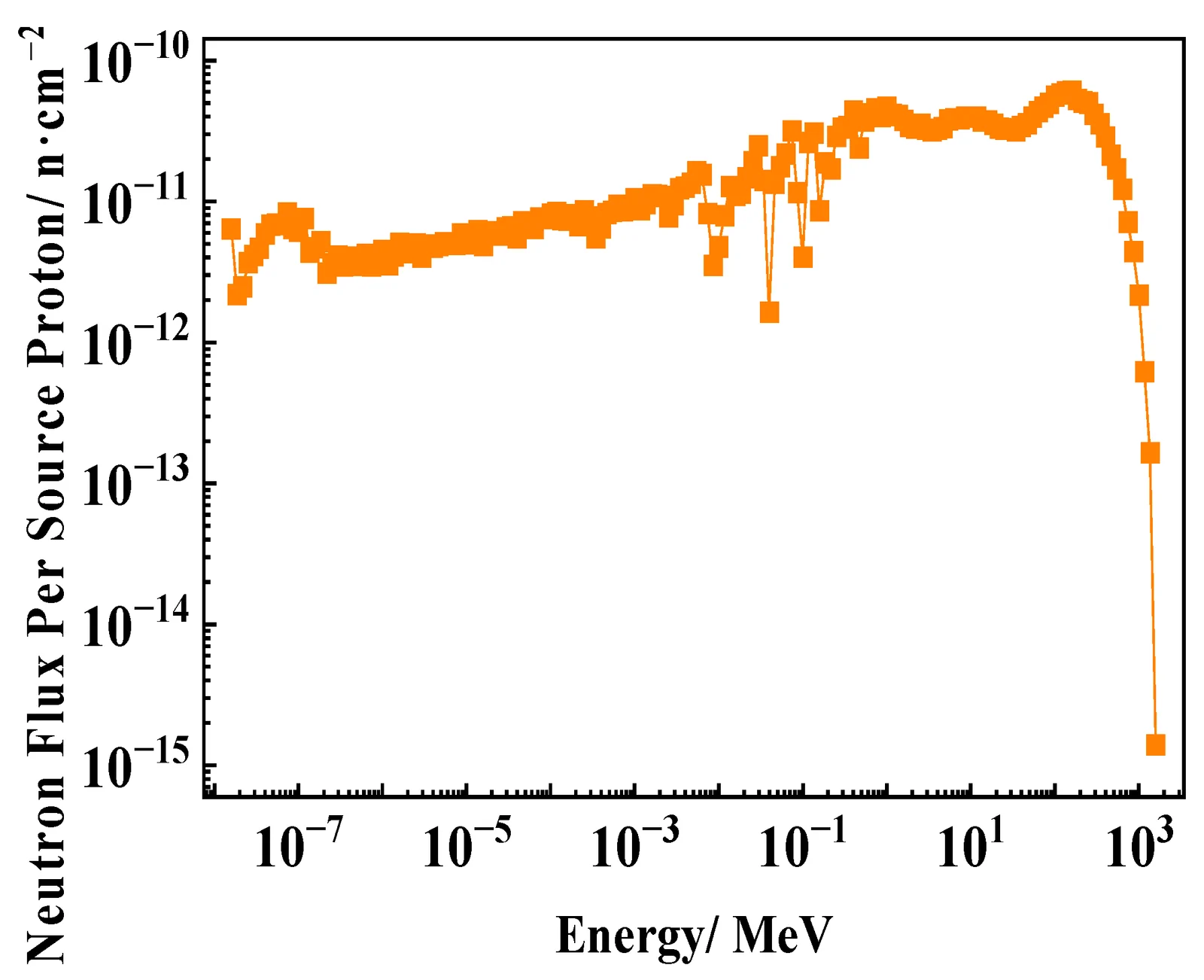
ANIS atmospheric neutron spectrum. The neutron flux was normalized per proton incident on the spallation target; therefore, it does not represent the absolute neutron flux rate during irradiation.

As shown in Figure 2, the neutron energy spectrum generated by this facility ranges from meV to GeV. The spectrometer features a high neutron flux and has been widely used to test the effects of radiation on electronic devices, demonstrating the ability to simulate real-world engineering conditions. The key features and specifications of the ANIS facility are summarized in Table 2. The devices were irradiated with the full ANIS neutron spectrum ranging from meV to GeV, including thermal, epithermal, and fast neutrons. The reported cumulative neutron fluences were 7.0 × 10^10^, 1.0 × 10^11^, and 3.9 × 10^12^ n/cm^2^, respectively, as monitored for neutron energies above 1 MeV. These fluence levels were selected to compare the device responses under two relatively low accumulated-fluence conditions and one substantially higher accumulated-fluence condition, thereby covering both the early-stage response and a clearly measurable degradation regime. They were not intended to represent direct mission-equivalent fluences for a specific space orbit or mission duration. To obtain the required neutron fluence rate for each irradiation condition, the collimator setting was adjusted, and the beam spot size was reduced from 10 × 10 cm^2^ to 2.5 × 2.5 cm^2^.

Secondary photons may coexist with the neutron radiation field at the ANIS facility. During the present irradiation experiment, however, the gamma-ray absorbed dose at the device position was not independently monitored. In addition, owing to limitations of the experimental site, it was difficult to install and maintain a stable online biasing and monitoring system within the irradiation area. Therefore, the devices were irradiated under unbiased conditions, with no external bias voltage applied to any device pin. Accordingly, the results obtained in this study primarily reflect the changes in the electrical performance of the devices after cumulative neutron irradiation in the non-operating state. These results may provide a reference for evaluating the radiation reliability of this type of device during unbiased storage or under non-operating conditions, but they cannot directly represent its radiation response under actual powered operating conditions. After irradiation, the devices were transferred to a conventional testing environment for electrical characterization.

## 3. Results and Discussion

### 3.1. GaAs-LED Neutron Irradiation Experiment Results

**Figure 3:**
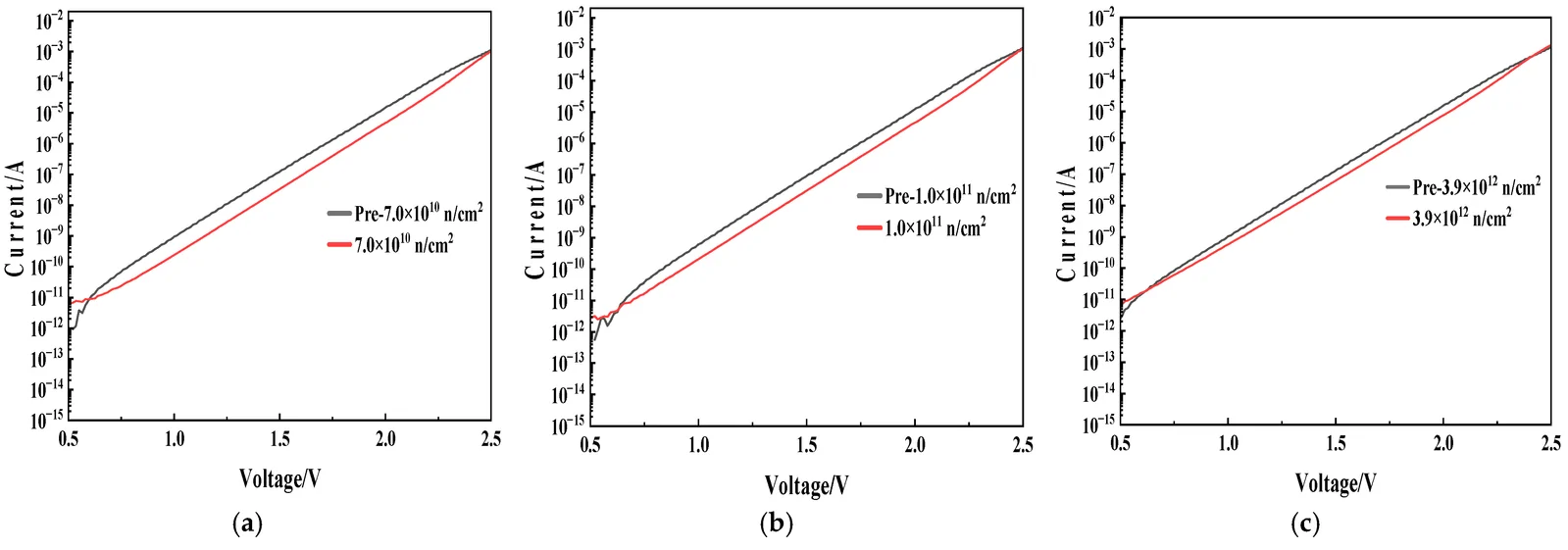
Forward electrical characteristics of the GaAs-LED: (**a**) 7.0 × 10^10^ n/cm^2^; (**b**) 1.0 × 10^11^ n/cm^2^; (**c**) 3.9 × 10^12^ n/cm^2^.

**Figure 4:**
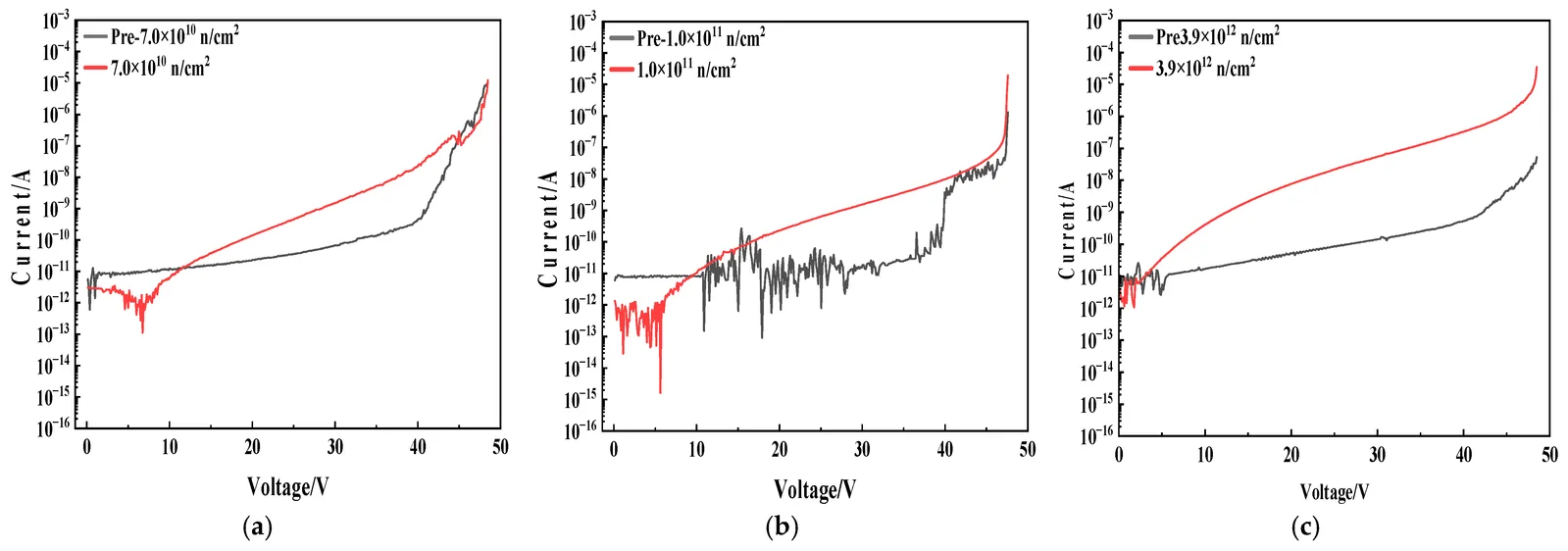
Reverse electrical characteristics of the GaAs-LED: (**a**) 7.0 × 10^10^ n/cm^2^; (**b**) 1.0 × 10^11^ n/cm^2^; (**c**) 3.9 × 10^12^ n/cm^2^.

Figure 3 and Figure 4 show the changes in the forward and reverse electrical characteristics of the GaAs-LED in the optical MOS solid-state relay after neutron irradiation. Within the neutron fluence range investigated in this study, the forward I–V curves of the irradiated samples remained generally similar and did not exhibit any pronounced variation with increasing neutron fluence. The forward current of the GaAs-LED is mainly composed of the minority-carrier diffusion current in the quasi-neutral regions and the recombination current in the space-charge region [20] and can be expressed as follows:

(1) $$I_{LED}=I_{\mathrm{diff}}+I_{SCR}$$

Carrier recombination in the space-charge region mainly occurs through two pathways: radiative recombination and nonradiative recombination. Radiative recombination determines the optical output power, whereas nonradiative recombination determines the forward current [21]. Displacement defects induced by neutron irradiation may act as nonradiative recombination centers, thereby affecting the minority-carrier lifetime as well as the radiative and nonradiative recombination processes. However, changes in these internal recombination processes do not necessarily produce responses of comparable magnitude in the terminal forward current of the device. Therefore, the results of this study indicate that, within the investigated neutron fluence range and under the present test conditions, the forward electrical characteristics of the GaAs-LED did not exhibit a pronounced dependence on neutron fluence.

In contrast, the reverse electrical characteristics of the GaAs-LED exhibit a clear dependence on neutron fluence. Specifically, the reverse current rises progressively with increasing fluence, and the change becomes more pronounced at higher fluences.

The increase in reverse current is attributed to displacement defects induced by neutron irradiation in the GaAs-LED side, which form defect energy levels within the material’s bandgap [22,23]. These defect levels act as effective non-radiative generation–recombination (GR) centers, significantly enhancing the electron–hole pair generation–recombination rate and consequently increasing the device’s reverse current [22,24,25].

### 3.2. MOSFET Neutron Irradiation Experiment Results and Simulation

The off-state leakage current, which can be directly measured through the external terminals of the device, was selected as the characterization parameter to evaluate the effect of neutron irradiation on the electrical performance of the output stage.

The off-state leakage current is defined as the leakage current measured between the source and drain terminals when the input voltage of the optical MOS solid-state relay is zero, the input-side GaAs-LED is not emitting light, and the output-side power MOSFET is in the off state. Since the optical MOS solid-state relay investigated in this study adopts a bidirectional MOSFET output structure, the source and drain terminals of the MOSFET can be interchanged under different biasing directions. Therefore, one MOSFET direction was selected for the leakage current measurement in this work. Specifically, Pin 5 was connected to the high-voltage bias terminal and served as the drain terminal of the tested MOSFET, while Pin 6 was connected to the low-potential reference terminal and served as the source terminal.

During the measurement, a drain-source voltage sweep from 0 to 50 V was applied between Pin 5 and Pin 6. A total of 401 voltage sampling points were configured during the sweep, and the corresponding stable leakage current value was recorded at each voltage point. The obtained current–voltage data were used to characterize the off-state I–V characteristics of the output-side MOSFET.

**Figure 5:**
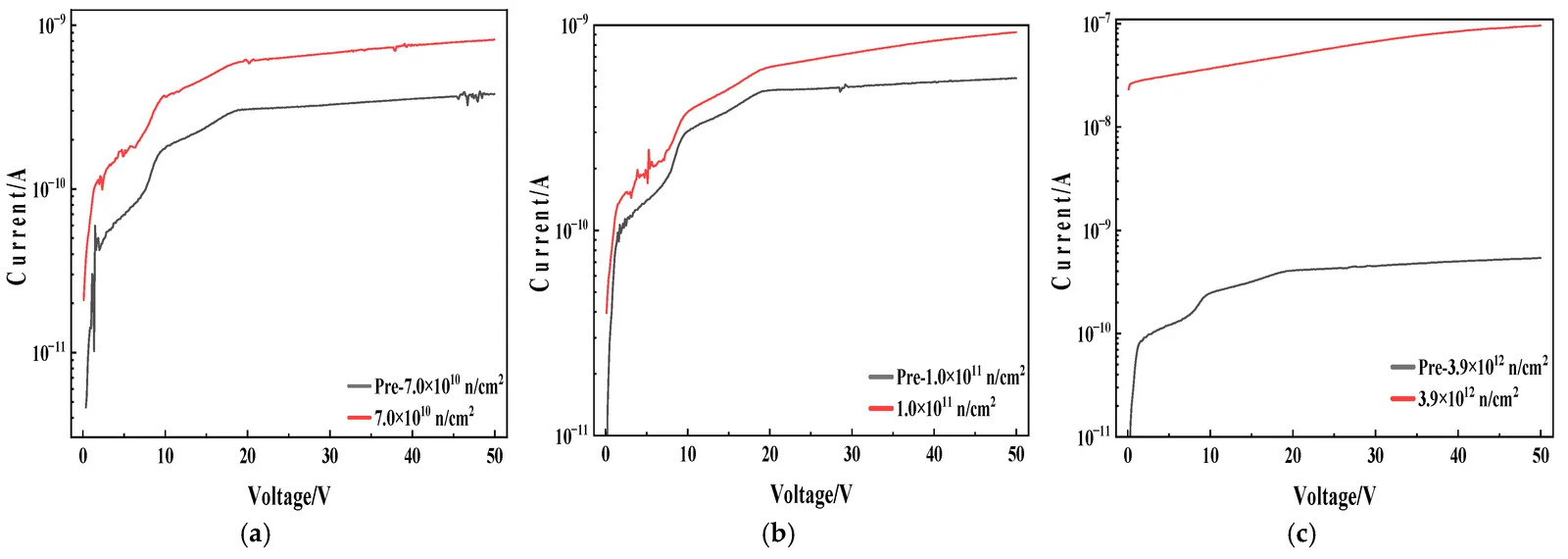
Variation in MOS off-state leakage current under different neutron fluences: (**a**) 7.0 × 10^10^ n/cm^2^; (**b**) 1.0 × 10^11^ n/cm^2^; (**c**) 3.9 × 10^12^ n/cm^2^.

Figure 5 presents the measured off-state leakage current of the output-side MOSFET after neutron irradiation. The results show that, over the neutron fluence range investigated in this study, from 7.0 × 10^10^ n/cm^2^ to 3.9 × 10^12^ n/cm^2^, the off-state leakage current exhibited an overall increasing trend. The difference in off-state leakage current between the fluences of 7.0 × 10^10^ and 1.0 × 10^11^ n/cm^2^ was relatively small, whereas a pronounced increase was observed when the neutron fluence reached 3.9 × 10^12^ n/cm^2^.

Based on existing studies, MOSFET devices are known to be insensitive to displacement damage effects [26]. However, in this experiment, a clear correlation was still observed between the off-state leakage current of the output-side power MOSFET in the optical MOS relay and the neutron fluence, which is inconsistent with existing understanding of neutron irradiation effects in MOSFETs. Unlike MOSFETs, photodiodes are relatively sensitive to displacement damage, and their dark current increases significantly after irradiation [27,28]. The increase in photodiode dark current affects the gate voltage of the output-side power MOSFET, thereby influencing its off-state leakage current.

To further investigate the possible coupling mechanism described above, Sentaurus TCAD was employed to perform a qualitative analysis of this process. Because the optical MOS solid-state relay investigated in this study is a commercial device, only limited information regarding its internal structure was available. According to information confirmed by the manufacturer, the input-side light-emitting unit is a GaAs-based LED, whereas the internal photodiode and the output-side power MOSFET are both Si-based devices. However, detailed information concerning the internal geometrical dimensions, doping profiles, junction depths, and fabrication processes of the actual device is proprietary to the manufacturer and was therefore unavailable. Accordingly, the TCAD model developed in this study was not intended to precisely reproduce the actual internal structure of the commercial device but rather to analyze the variation trends of the relevant electrical parameters and their possible underlying physical mechanisms.

In the simulation, only a Si-based photodiode device model was constructed to represent the internal photovoltaic conversion unit. Because the input-side GaAs-LED is optically coupled to but electrically isolated from the output-side circuit, a complete GaAs-LED device structure was not modeled. Instead, photogenerated carriers were introduced into the photodiode using the Optical model in Sentaurus Device to simulate the process by which the photodiode receives the optical signal emitted by the input-side LED and generates a photocurrent. The light emitted by the LED was therefore treated as the incident optical condition applied to the Si-based photovoltaic conversion unit. Regarding the physical models, the simulations simultaneously solved the Poisson equation and the electron and hole continuity equations while accounting for carrier drift–diffusion transport, carrier mobility, and Shockley–Read–Hall generation–recombination processes.

**Figure 6:**
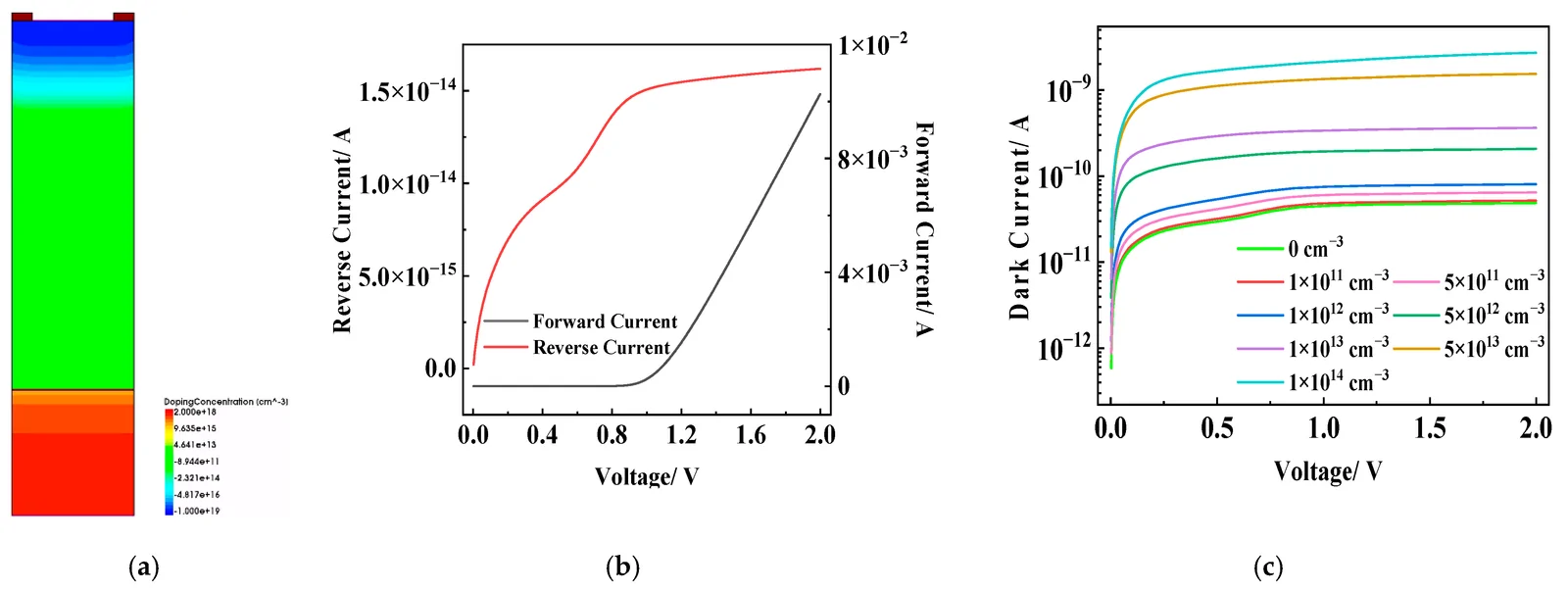
(**a**) Photodiode simulation model; (**b**) photodiode electrical characteristic curve; (**c**) dark current of the photodiode as a function of defect concentration.

Figure 6a shows the typical Si-based photodiode device structure established in this paper. This structure primarily consists of a PN junction active region, an electrode contact region, and the corresponding semiconductor substrate region. Figure 6b presents the macroscopic electrical characteristic curves of the photodiode obtained from simulations based on this structure. The simulation results indicate that this structure effectively reflects the fundamental electrical behavior of the photodiode within an optical MOS solid-state relay.

Figure 6c shows the simulated electrical characteristics obtained by introducing defect levels generated by displacement damage induced by neutron irradiation into the bulk region of a photodiode and varying the defect concentration in the bulk region to simulate different damage levels under varying neutron fluences. The simulation results indicate that as the defect concentration gradually increases, the dark current of the Si-based photodiode under reverse bias conditions increases significantly. This result suggests that neutron-induced displacement damage significantly degrades the dark current characteristics of the photodiode, thereby causing a change in the gate voltage of the output-side power MOSFET.

In an optical MOS solid-state relay, the operating state of the photodiode directly determines the conduction behavior of the output MOSFET. When the photodiode receives the optical signal emitted by the LED, incident photons are absorbed in the PN junction and its adjacent depletion region, generating electron-hole pairs. These pairs are separated under the influence of the electric field established in the junction region, thereby generating a photocurrent. The generated photocurrent charges the equivalent capacitance of the MOSFET’s gate, causing the gate voltage to gradually increase. When the gate voltage exceeds the MOSFET’s threshold voltage, both output MOSFETs turn on. The optical MOS solid-state relay transitions from the off state to the on state.

**Figure 7:**
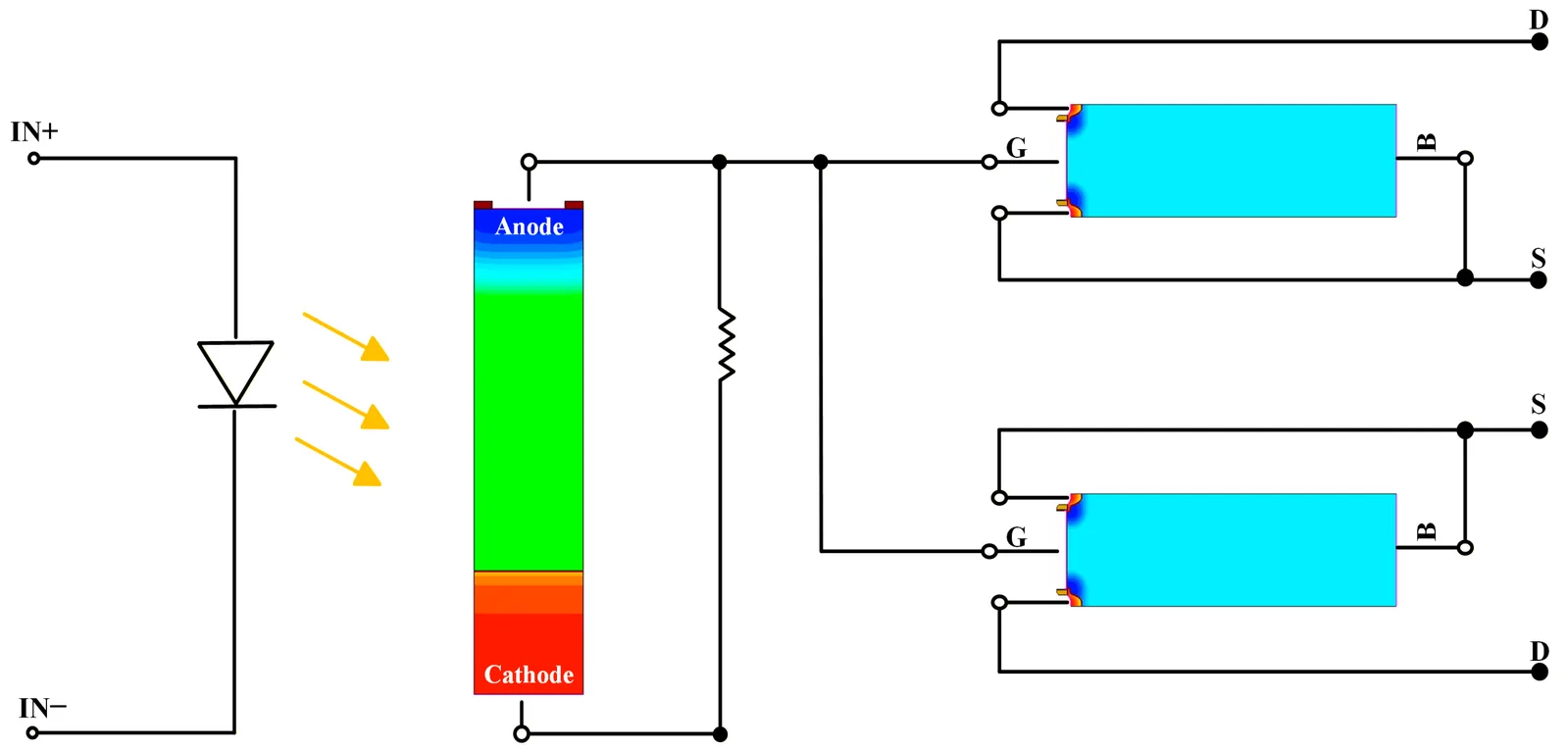
TCAD simulation circuit schematic.

In this study, the Mixed-mode simulation method in Sentaurus TCAD was employed to connect a typical Si-based photodiode with the output-side Si-based power MOSFET at the circuit level, thereby constructing an equivalent device-circuit mixed-mode simulation model of the optical MOS solid-state relay, as shown in Figure 7. Since the detailed internal structure, process parameters, and key electrical parameters of the commercial optical MOS solid-state relay are unavailable, the established model is not intended to fully reproduce the actual internal structure and all parasitic parameters of the commercial device. Instead, it is mainly used to analyze the coupling relationship between neutron irradiation-induced photodiode degradation and the electrical performance variation in the output-side MOSFET.

In this mixed-mode model, the Optical model in Sentaurus Device was employed to introduce the optical excitation generated by the GaAs-based LED. In addition, an equivalent resistor was introduced between the photodiode output node and the MOSFET gate node. It should be noted that this resistor does not represent an actual discrete resistor component existing inside the commercial optical MOS solid-state relay but rather represents the equivalent charge–discharge path of the MOSFET gate node. During device operation, the current generated by the photodiode charges the equivalent parasitic capacitance of the MOSFET gate, thereby modifying the gate node potential. When the optical signal disappears or the photodiode output current changes, the stored gate charge is released through the internal equivalent discharge path, causing the gate potential to gradually recover. Therefore, this equivalent resistor is mainly introduced to describe the dynamic charge release behavior of the MOSFET gate node and the coupling relationship between the photodiode output node and the MOSFET gate.

It should be particularly emphasized that the focus of this work is not the operation of the optical MOS solid-state relay under normal light-driven turn-on conditions but rather the degradation mechanism of the device off-state electrical characteristics under neutron irradiation. When the input-side GaAs-LED is not emitting light, neutron irradiation induces displacement damage defects in the Si-based photodiode, resulting in an increase in dark current. This increased dark current can affect the charging and discharging behavior of the equivalent MOSFET gate capacitance through the internal coupling path, thereby modifying the gate node potential and further increasing the off-state leakage current of the output-side MOSFET. Therefore, the mixed-mode model is mainly used to analyze the physical coupling process of “photodiode radiation-induced degradation–gate node potential disturbance–MOSFET off-state leakage current variation.”

The value of this equivalent resistor affects the transient coupling behavior between the photodiode output node current and the MOSFET gate voltage. However, in the present simulation, this parameter was assumed to remain unchanged before and after neutron irradiation. This assumption was made because this parameter represents an equivalent charge–discharge path rather than an actual radiation-sensitive semiconductor structure. Furthermore, previous radiation effects studies have shown that, compared with the significant electrical degradation caused by defect generation, recombination, and trapping processes in semiconductor devices, conventional passive components generally exhibit much lower radiation sensitivity [29,30]. Therefore, under the irradiation conditions investigated in this work, the influence of variations in this equivalent parameter on the overall device degradation response is considered negligible.

To further analyze the effect of neutron-induced displacement damage on the performance degradation of optical MOS solid-state relays, neutron displacement damage effects were equivalently simulated in this study by introducing bulk defects into the TCAD model based on the defect parameters listed in Table 3 [31]. Based on the experimentally measured macroscopic electrical characteristics of irradiated Si-based detectors, the referenced study established a multilevel defect model suitable for analyzing radiation damage in Si and provided key parameters, including defect energy levels, electron and hole capture cross-sections, and defect introduction rates. In that study, these defect parameters were incorporated into Sentaurus TCAD, and the model was calibrated and validated through comparison with experimental results obtained from irradiated devices. In the present work, this radiation-defect model was employed in TCAD simulations to qualitatively analyze the effects of neutron irradiation on the electrical characteristics of the output-side power MOSFET, thereby providing physical support for the proposed internal coupling mechanism.

**Figure 8:**
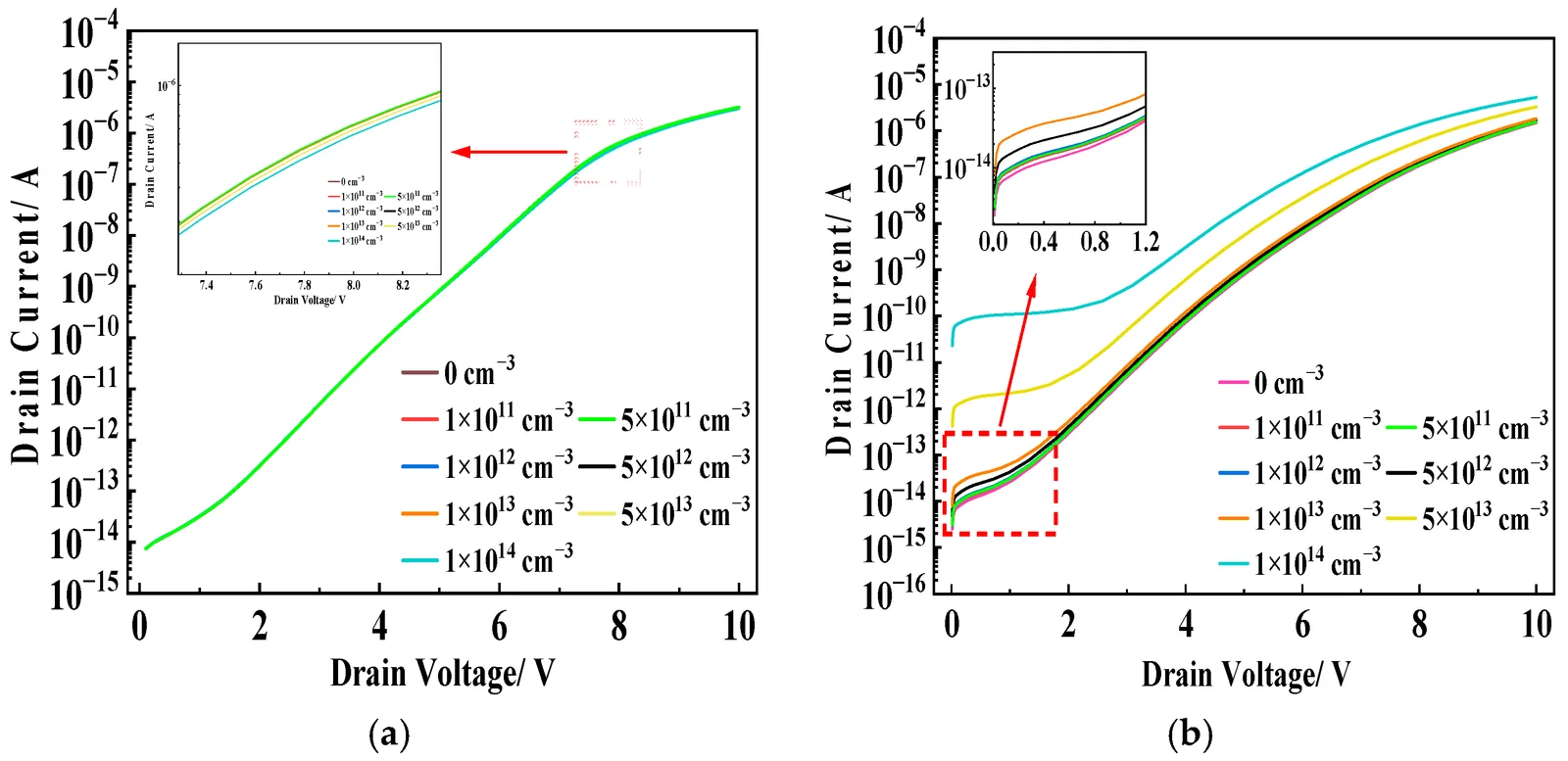
Curves showing the variation in MOSFET off-state leakage current with defect concentration: (**a**) independent simulation of MOSFET off-state leakage current; (**b**) joint simulation of MOSFET off-state leakage current.

Figure 8a shows the simulation results of the off-state leakage current in a single MOSFET device after the introduction of bulk defects. It can be seen that as the bulk defect concentration increases, the MOSFET off-state leakage current does not change significantly. When only displacement damage defects within the MOSFET device are considered, their effect on the off-state leakage current is weak, making it difficult to explain the experimental observation that the off-state leakage current increases with increasing neutron dose.

Figure 8b presents the results obtained from the device-circuit mixed-mode simulation of the photodiode and MOSFET. The simulation results indicate that, as the bulk defect concentration increases, the MOSFET off-state leakage current exhibits an upward trend overall. This behavior can be explained by the enhanced carrier generation caused by displacement-damage defects of the photodiode, thereby increasing the photodiode dark current. The increased dark current further produces a weak charging effect on the equivalent gate capacitance of the output-side power MOSFET, leading to an increase in the gate voltage and consequently an increase in the leakage current of the MOSFET under the off-state condition. This mixed-mode simulation result is consistent with the trend observed in the neutron irradiation experiments, further indicating that the increase in photodiode dark current and its modulation effect on the MOSFET gate bias are important factors responsible for the increase in the off-state leakage current of the output side. The present experimental and simulation results support the displacement-damage-induced photodiode–MOSFET coupling mechanism as an important explanation for the increase in off-state leakage current. Nevertheless, because the photon absorbed dose was not independently measured, a possible contribution from photon-induced ionizing damage in the output MOSFET cannot be quantitatively excluded.

### 3.3. Annealing Experiment Results and Discussion

To further investigate the recovery characteristics of the devices after neutron irradiation, the samples in each group were subjected to annealing treatments following the irradiation experiments. To characterize the recovery of the electrical parameters with annealing time under different neutron fluence conditions, the annealing recovery ratio, R(t), was defined as follows:

(2) $$R(t)=\frac{I_{irr}-I(t)}{I_{irr}-I_{pre}}\times 100\%$$

where I_pre_ is the current before irradiation, I_irr_ is the current after irradiation, and I(t) is the current measured after an annealing time t. A larger R(t) indicates a greater degree of recovery of the electrical parameter toward its pre-irradiation value.

The annealing experiment primarily consists of two parts: room-temperature annealing, where samples are left at room temperature, and rapid high-temperature annealing, conducted in a high-temperature annealing chamber. During room-temperature annealing, the room temperature was maintained at 17 °C. To demonstrate the effectiveness of long-term annealing at room temperature and to evaluate the stability of the devices under normal storage conditions, we began testing the room-temperature-annealed samples on the 30th day of static storage, with static electrical property measurements performed every 3 days thereafter. After 36 days of room-temperature testing, the samples were subjected to rapid high-temperature annealing. The temperature of the high-temperature annealing furnace was set to 200 °C, with annealing durations of 2 h, 4 h, 8 h, 10 h, and 20 h, respectively.

This test involved annealing devices exposed to three distinct neutron dose levels. Based on irradiation test results, significant trends in electrical performance before and after irradiation were analyzed. The primary electrical performance parameters measured after annealing were the reverse electrical performance of the input-stage GaAs-LED and the off-state leakage current of the output-stage power MOSFET.

#### 3.3.1. Annealing Behavior of GaAs-LED Side

**Figure 9:**
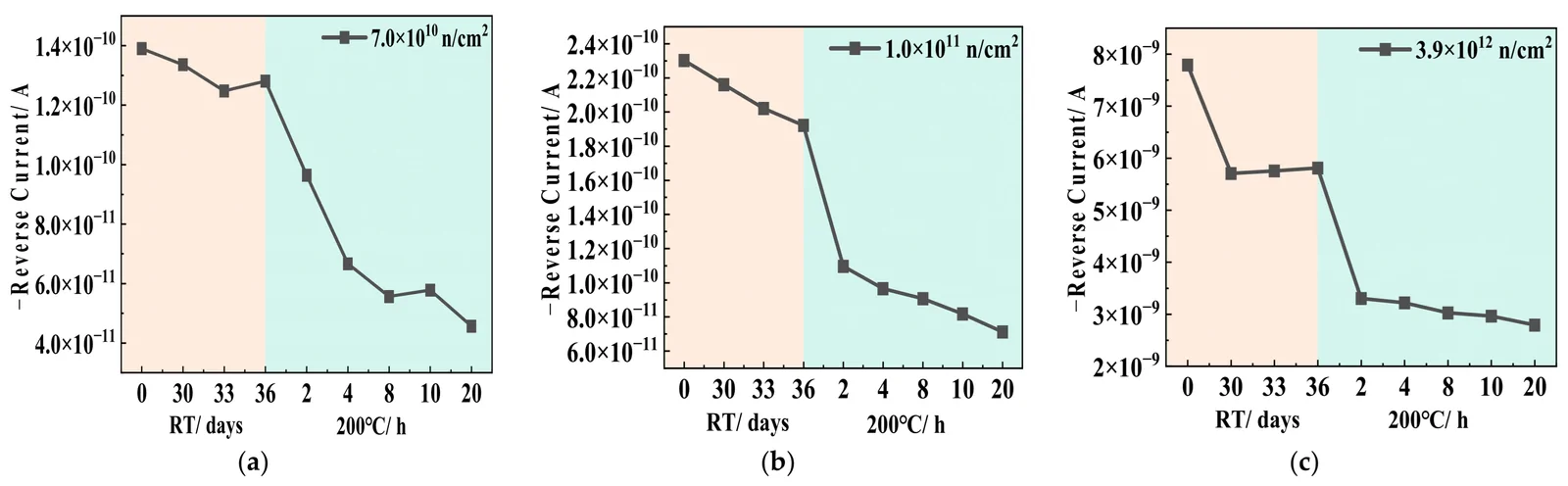
Relationship curve between reverse current annealing behavior and time for the LED side: (**a**) 7.0 × 10^10^ n/cm^2^; (**b**) 1.0 × 10^11^ n/cm^2^; (**c**) 3.9 × 10^12^ n/cm^2^.

Figure 9 and Table 4 present the variation in the GaAs-LED reverse current with annealing time and the corresponding annealing recovery ratio, R(t), respectively. During room-temperature annealing, the recovery of the reverse current was relatively limited. After 30 days of room-temperature annealing, the recovery ratios of the samples irradiated at neutron fluences of 7.0 × 10^10^, 1.0 × 10^11^, and 3.9 × 10^12^ n/cm^2^ were 5.01%, 7.13%, and 26.85%, respectively. When the room-temperature annealing duration was further extended to 33 and 36 days, the recovery ratios of all samples exhibited only minor fluctuations. Thus, during the room-temperature annealing period from 30 to 36 days, the recovery of the GaAs-LED reverse current remained limited, and no pronounced additional annealing effect was observed.

After the samples were subsequently subjected to high-temperature annealing, the recovery of the reverse current became substantially more pronounced. After 2 h of high-temperature annealing, the recovery ratios of the samples under the three neutron fluence conditions increased to 39.49%, 60.54%, and 57.80%, respectively. With increasing high-temperature annealing time, the recovery ratios exhibited an overall increasing trend and reached 86.60%, 79.87%, and 64.40%, respectively, after 20 h. Correspondingly, the GaAs-LED reverse current gradually decreased with increasing high-temperature annealing time. These results indicate that increasing the annealing temperature can significantly promote the recovery of neutron-induced defects, suggesting that the defect recovery process is thermally activated. As the annealing time increased, the recovery rate gradually decreased, and the reverse current tended toward a relatively stable level.

The observed annealing behavior can be attributed to the thermal evolution of neutron-induced displacement damage defects in the GaAs-based LED. The radiation-induced defects can act as generation–recombination centers and enhance carrier recombination processes, thereby degrading carrier transport properties and increasing the reverse leakage current. During the annealing process, some unstable point defects and defect complexes may undergo thermally activated migration, recombination, or structural transformation, leading to partial recovery of the electrical characteristics of the device. The limited recovery observed during room-temperature annealing indicates that the dominant radiation-induced defects in GaAs exhibit relatively high thermal stability at room temperature. In contrast, high-temperature annealing provides sufficient thermal energy to promote the effective migration and recombination of some defects, thereby facilitating the recovery of device performance.

#### 3.3.2. Annealing Behavior of MOSFET Side

**Figure 10:**
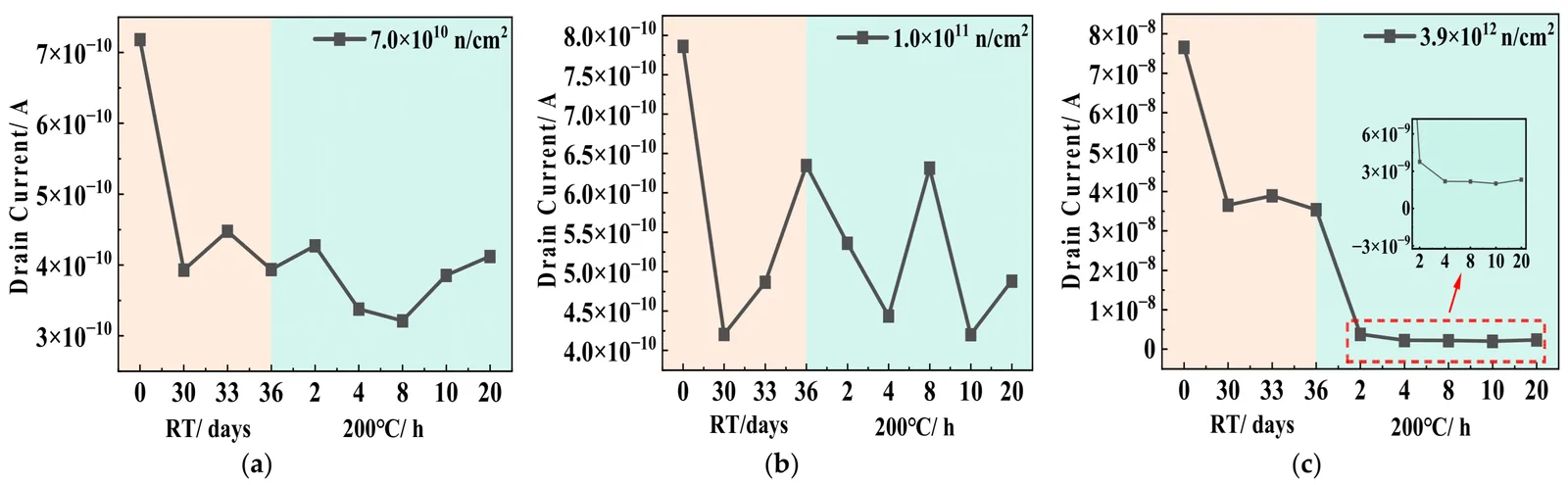
Relationship curve between MOSFET-side off-state leakage current annealing behavior and time: (**a**) 7.0 × 10^10^ n/cm^2^; (**b**) 1.0 × 10^11^ n/cm^2^; (**c**) 3.9 × 10^12^ n/cm^2^.

Figure 10 shows the variation in the off-state leakage current of the output-side power MOSFET with annealing time. Compared with the annealing results of the GaAs-LED reverse current shown in Figure 9, the annealing response of the MOSFET-side off-state leakage current was relatively weak and exhibited poorer regularity. At neutron fluences of 7.0 × 10^10^ and 1.0 × 10^11^ n/cm^2^, the irradiation-induced variation in the off-state leakage current was relatively small. During annealing, the measured leakage current exhibited only limited fluctuations and did not show a systematic dependence on annealing time. Therefore, under these two neutron fluence conditions, the annealing recovery of the MOSFET-side off-state leakage current was not pronounced.

In contrast, at the highest neutron fluence of 3.9 × 10^12^ n/cm^2^, the off-state leakage current of the power MOSFET exhibited a clear annealing response. Therefore, the annealing recovery ratio, R(t), was calculated for this fluence condition, as shown in Table 5. After 30 days of room-temperature annealing, the recovery ratio was 52.58%. When the room-temperature annealing duration was extended to 33 and 36 days, the recovery ratios were 49.47% and 54.11%, respectively, indicating small fluctuations rather than a monotonic recovery trend. After high-temperature annealing was introduced, the recovery became substantially more pronounced. Following 2 h of high-temperature annealing, the recovery ratio rapidly increased to 95.65%. With further annealing for 4, 8, and 10 h, the recovery ratios reached 97.71%, 97.72%, and 97.94%, respectively. After 20 h, the recovery ratio decreased slightly to 97.54% because of a small rebound in the measured leakage current. Overall, the recovery ratio remained within approximately 97.5–97.9% after 4 h of high-temperature annealing, suggesting that most of the recoverable component of the irradiation-induced leakage-current degradation was annealed during the initial high-temperature treatment and that the recovery subsequently approached a relatively stable level.

The annealing behavior of the output-side MOSFET can be attributed to the thermal evolution of neutron-induced defects in the Si-based semiconductor materials. Neutron irradiation can introduce displacement damage defects in silicon, including vacancies, divacancies, and vacancy-related defect complexes. These defects can introduce deep energy levels within the silicon bandgap and affect the leakage characteristics of semiconductor devices by influencing carrier generation, recombination, and trapping processes. The rapid recovery observed after high-temperature annealing further indicates that the dominant recoverable degradation component is associated with thermally activated defect processes.

## 4. Conclusions

By integrating the results of neutron irradiation experiments, device–circuit co-simulations, and annealing experiments, this study systematically investigated the irradiation-induced degradation correlations among the different functional units of optical MOS solid-state relays. The experimental results show that the input-side GaAs-LED is relatively sensitive to neutron irradiation, with its reverse leakage current increasing with neutron fluence. The off-state leakage current of the output-side power MOSFET also increases as the neutron fluence increases. However, this phenomenon cannot be adequately explained solely by displacement damage within the MOSFET itself. Based on a device–circuit co-simulation model established using the Mixed-Mode method in Sentaurus TCAD, this study proposes an internal coupled degradation mechanism: neutron-induced displacement damage alters the dark current of the internal photodiode, and this change is coupled to the MOSFET gate node through the internal circuitry, thereby affecting the off-state electrical characteristics of the output stage. The annealing results show that the GaAs-LED exhibits a relatively pronounced recovery response, whereas the annealing recovery of the output-side off-state leakage current is comparatively weak and does not show a clear trend at lower neutron fluences.

The practical significance of this study lies in demonstrating that the radiation response of an optical MOS solid-state relay may not be determined solely by the degradation of an individual functional unit but may instead be jointly influenced by the coupling among the input light-emitting unit, the internal photovoltaic conversion unit, and the output power device. Therefore, radiation reliability assessment and radiation-hardening design for this type of device should comprehensively consider the degradation of the internal photovoltaic conversion performance and its influence on the operating state of the output stage. The analytical approach proposed in this study may provide a reference for optically isolated power-switching devices with similar internal structures and operating principles, while also offering experimental evidence and a physical basis for failure analysis and targeted radiation-hardening design.

Several limitations of this study should nevertheless be acknowledged. Only one device model from a single manufacturer was investigated, and the number of samples under each irradiation condition was limited. Therefore, the present results cannot be directly generalized to all commercial optical MOS solid-state relays. In addition, no external bias voltage was applied to any device pin during irradiation. Consequently, the results mainly reflect the cumulative neutron irradiation response of the devices in the non-operating state and cannot represent their radiation behavior under actual powered operating conditions. Furthermore, because detailed information on the internal geometry, doping profiles, and other parameters of the commercial device was unavailable, the present simulations were primarily used to verify the physical plausibility of the proposed coupling mechanism and are insufficient to quantitatively determine its specific contribution to the variation in the off-state leakage current of the actual device. Future studies should broaden the range of device models and manufacturers investigated and increase the number of replicate samples under identical irradiation conditions. Neutron irradiation experiments should also be conducted under different input- and output-bias conditions, together with online monitoring, to investigate the effects of the operating electric field on defect evolution, photovoltaic conversion efficiency, and output-side failure behavior. These efforts will contribute to the further development of radiation-hardening design strategies and more reliable in-service reliability assessments for optical MOS solid-state relays.

## Figures and Tables

**Table 1:** Relevant parameter information of the experimental device.

Device Model	Input Current/mA	DC Output Current/A	DC Output Voltage/V
JGW-QY2M	5–20	5	60

**Table 2:** Key features and specifications of ANIS [18].

Indicators	Specifications
Neutron Spectrum	Coverage range: meV-GeV, energy spectrum is the closest to JEDEC spectrum [19]
Collimating Spot Size	10 cm × 10 cm, 5 cm × 5 cm, 2.5 cm × 2.5 cm, 1.0 cm × 1.0 cm
Beam Expansion Spot Size	65 cm × 65 cm, continuous expansion to 80 cm × 80 cm; 40 cm × 40 cm
Neutron Flux	Collimated beam spot: approximately 10^5^~10^7^ n/cm^2^/s, adjustable over 5 discrete intensity levels; Beam expansion spot: approximately 10^3^~10^7^ n/cm^2^/s, adjustable using the neutron flux controller

**Table 3:** Defect parameters formed by 1 MeV equivalent neutrons in bulk silicon [31].

Level	Ass	σ_n_/cm^2^	σ_p_/cm^2^
Ec−0.42 eV	V-V^(−/0)^	2 × 10^−15^	1.2 × 10^−14^
Ec−0.50 eV	V-V-O^(−/0)^	5 × 10^−15^	3.5 × 10^−14^
Ev+0.36 eV	C_i_-O_i_	2.0 × 10^−18^	2.5 × 10^−15^

**Table 4:** Annealing recovery rate R(t) of the GaAs-LED reverse current.

Annealing Time	7.0 × 10^10^ n/cm^2^	1.0 × 10^11^ n/cm^2^	3.9 × 10^12^ n/cm^2^
0 d	0%	0	0
30 d	5.01%	7.13%	26.85%
33 d	13.17%	14.12%	26.20%
36 d	10.11%	19.09%	25.49%
2 h	39.49%	60.54%	57.80%
4 h	67.09%	67.16%	58.89%
8 h	77.33%	70.05%	61.40%
10 h	75.32%	74.59%	62.19%
20 h	86.60%	79.87%	64.40%

**Table 5:** Annealing recovery rate of the power MOSFET off-state leakage current.

Annealing Time	3.9 × 10^12^ n/cm^2^
0 d	0
30 d	52.58%
33 d	49.47%
36 d	54.11%
2 h	95.65%
4 h	97.71%
8 h	97.72%
10 h	97.94%
20 h	97.54%

## Data Availability

The data presented in this study are available upon request from the corresponding author.
